# Potential ecological suitability of *Epimedium sagittatum* in China under climate change based on an optimized MaxEnt model

**DOI:** 10.3389/fpls.2026.1881558

**Published:** 2026-07-15

**Authors:** Kang Ju, Mengya Fu, Lina Liu, Penghui Liu, Bing He

**Affiliations:** 1 Bozhou Institute, Anhui Provincial Academy of Chinese Medicine, Bozhou, Anhui, China; 2 BoZhou Vocational and Technical College, Bozhou, China; 3 College of Medicine and Health Care, Fangchenggang Vocational and Technical College, Fangchenggang, Guangxi, China; 4 College of Clinical Medicine, Qinghai University, Xining, Qinghai, China

**Keywords:** climate change, environmental variables, *Epimedium sagittatum*, MAXENT model, potential suitable habitat prediction

## Abstract

*Epimedium sagittatum* is an important tonic medicinal plant rich in prenylated flavonoids represented by icariin, and its wild resources are affected by harvesting pressure, habitat change, and climate change. Based on 160 spatially thinned occurrence records from CVH and GBIF and 103 candidate environmental variables, this study used an optimized MaxEnt model to evaluate the current and future potential ecological suitability of *E. sagittatum* in China. After variable screening, 15 predictors were retained. Kuenm parameter tuning indicated that the optimal model used a regularization multiplier of 3.2 and a feature combination of linear, product, and threshold features. The optimized model had a training AUC of 0.9597 and a test AUC of 0.9475 +/- 0.0095. The cumulative contribution of minimum temperature of the coldest month (bio_6), May solar radiation (srad_05), November precipitation (prec_11), and temperature seasonality (bio_4) was 80.8%. Under current climatic conditions, the total suitable area was 1.38 × 10^6^ km^2^, mainly concentrated in Hunan, Hubei, Jiangxi, Anhui, Zhejiang, and adjacent humid regions. Future projections were adjusted to the 2050s and 2090s and compared under SSP1-2.6, SSP2-4.5, and SSP5-8.5. Total suitable area ranged from 1.37 × 10^6^ to 1.51 × 10^6^ km^2^ in the 2050s and from 1.41 × 10^6^ to 1.43 × 10^6^ km^2^ in the 2090s. Centroids shifted 22–55 km relative to the current centroid, generally toward the north but without a monotonic trajectory. These results provide a spatial reference for resource conservation, field surveys, and cultivation-zoning planning of *E. sagittatum*.

## 1 Introduction

Global climate change has become one of the major challenges faced by terrestrial ecosystems in the 21st century. The latest assessment of the Intergovernmental Panel on Climate Change (IPCC) indicates that, accompanied by the continuous rise in global temperature, the changes in precipitation patterns and the increasing frequency of extreme weather events are significantly affecting the growth, development and geographical distribution of plants (Parmesan and Yohe, 2003; Applequist et al., 2020). The growth of medicinal plants tends to have stricter requirements on climatic conditions. Climate change not only affects the distribution range and population size of the wild resources of medicinal plants, but also has a direct impact on the quality of medicinal materials and the accumulation of effective constituents, thereby influencing the stable supply of related medicinal material industries. Therefore, quantitatively predicting the potential distribution ranges of medicinal plants under future climate change is of great significance for scientifically formulating wild resource conservation strategies, planning artificial cultivation layouts, and safeguarding the sustainable development of the traditional Chinese medicinal materials industry.

To investigate the impacts of environmental change on species distribution, common ecological niche models currently include BIOCLIM, DOMAIN, GARP, GAM, GLM, ENFA and the Maximum Entropy model (MaxEnt) (Elith et al., 2006; Anderson, 2013). Among these, the MaxEnt model has been widely used in recent years for predicting the potential habitats of medicinal plants and assessing climate change impacts, owing to its good applicability in handling presence-only data and nonlinear relationships, as well as its ability to maintain high prediction accuracy under small-sample conditions (Phillips et al., 2006; Pearson et al., 2007; Elith et al., 2011; Merow et al., 2013). It has also been widely used in broader species-distribution and risk-prediction studies (Ma et al., 2021; Wan et al., 2020), and has been applied to medicinal plants such as *Angelica sinensis*, *Forsythia suspensa*, *Verbena officinalis*, *Cirsium lineare* and *Rubus idaeus*, with the corresponding results providing important support for the resource conservation and artificial cultivation zoning of these species (Shen et al., 2021; Zhang et al., 2018; Shi et al., 2023; Rong et al., 2024; Chen et al., 2025; Xi et al., 2025).

*Epimedium sagittatum* (Sieb. et Zucc.) Maxim., a perennial herbaceous plant of the genus *Epimedium* in the family Berberidaceae, is mainly distributed in the understory, forest margins and valley gullies of subtropical mountainous areas south of the Qinling Mountains in China. As one of the source plants of *Epimedii Folium* recorded in the *Pharmacopoeia of the People’s Republic of China*, *E. sagittatum* is used medicinally with its dried leaves, with a pungent and sweet taste, warm nature, and meridian tropism for the liver and kidney, exerting effects of tonifying kidney yang, strengthening tendons and bones, and dispelling wind-dampness; it is one of the important tonic traditional Chinese medicinal materials in China. Its leaves are rich in 8-prenylflavonoid constituents represented by icariin, with significant pharmacological and economic value in anti-osteoporosis, immune regulation and cardiovascular protection (Bi et al., 2022; Cheng et al., 2022; Wang et al., 2025). However, the supply of this medicinal material remains highly dependent on wild resources, and long-term over-harvesting and original habitat degradation have led to a continuous decline in its wild resources. To alleviate resource pressure, some regions have begun to attempt artificial introduction and cultivation; however, due to the lack of quantitative research on the ecological suitability of this species, site selection for cultivation often lacks scientific basis. In addition, the continuous changes in climate and ecological environment will inevitably affect its suitable habitat patterns. Therefore, using the MaxEnt model to investigate the key environmental factors influencing the suitable growth of *E. sagittatum* and their effects on suitable habitat distribution, and to predict its potential habitat under the context of climate change, is of great significance for the conservation, artificial cultivation and future sustainable development of this species.

Using China as the study region, this work compiled occurrence information from public specimen and biodiversity databases and combined climatic, topographic, vegetation, population, and soil variables. The Kuenm package was used to optimize MaxEnt parameters, and the potential suitable distribution of *E. sagittatum* under current and future climates was predicted. Suitable-area size, expansion and contraction patterns, and centroid migration were then analyzed under different climate pathways, providing spatial evidence for resource conservation, field surveys, and cultivation-zoning discussion.

## 2 Materials and methods

### 2.1 Collection and processing of occurrence data for *E. sagittatum*

**Figure 1:**
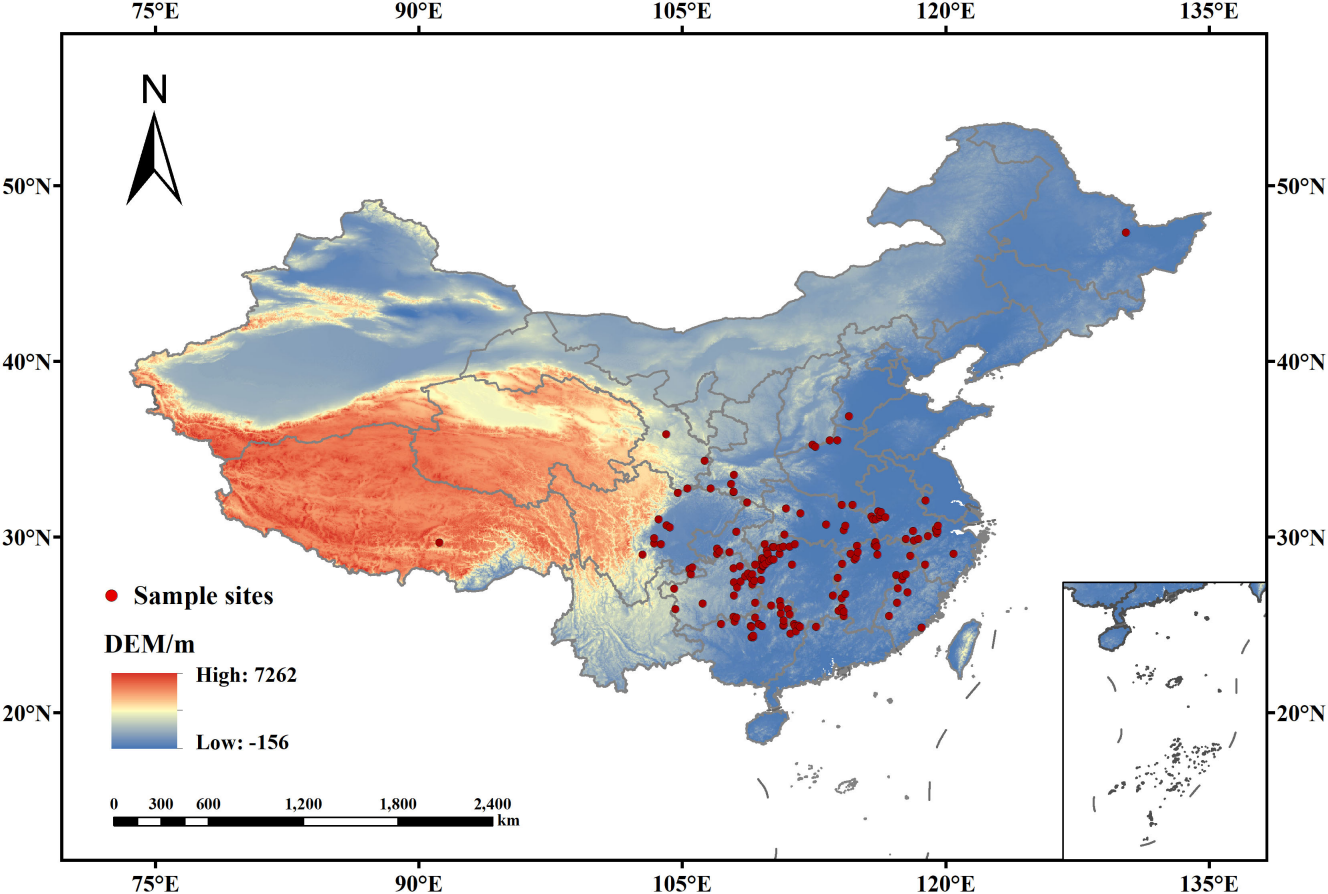
Spatial distribution of the 160 retained occurrence points of *Epimedium sagittatum* in China.

Occurrence data for E. sagittatum were obtained from two open databases: the Chinese Virtual Herbarium (CVH; https://www.cvh.ac.cn/) and the Global Biodiversity Information Facility (GBIF; https://www.gbif.org/; accessed 23 December 2025). To maintain temporal consistency between occurrence and climate data, only records after 1980 were collected. For specimens with locality descriptions but no coordinates, Google Earth was used for secondary checking and georeferencing. A total of 167 occurrence points were obtained after preliminary processing. To reduce the influence of spatial autocorrelation and sampling bias (Boria et al., 2014), ENMTools was used to remove duplicated or adjacent records in 5 km × 5 km grid cells, with only one valid point retained in each grid cell. Finally, 160 valid occurrence points were used for modelling (**Figure 1**).

### 2.2 Environmental data sources and processing

Current environmental data were obtained from WorldClim 2.1 (Hijmans et al., 2005; Fick and Hijmans, 2017) and included 19 bioclimatic variables, monthly precipitation, monthly maximum temperature, monthly minimum temperature, monthly mean temperature, and monthly solar radiation. The WorldClim database is available at https://worldclim.org/. Three topographic variables (altitude, slope, and aspect) were extracted from DEM data. Vegetation classification was obtained from the 1:1,000,000 Vegetation Map of China, population density from WorldPop (2020), and soil physicochemical properties from HWSD v1.2 (FAO/IIASA/ISRIC/ISS-CAS/JRC, 2012). Future climate data were based on CMIP6 and the Beijing Climate Center Climate System Model (BCC-CSM2-MR) (O'Neill et al., 2016; Wu et al., 2019), covering the 2050s (mean for 2041-2060) and the 2090s (mean for 2081-2100) under three Shared Socioeconomic Pathway scenarios: SSP1-2.6, SSP2-4.5, and SSP5-8.5. All environmental variables were resampled to the same 2.5 arc-min resolution (approximately 5 km) using bioclimatic variables as the reference grid. A total of 103 candidate environmental factors were obtained (**Table 1**).

**Table 1:** Grouped information for the candidate environmental variables used in the present study.

Variable code	Environmental factor	Variable code	Environmental factor
srad 1-12	Solar radiation	pop2020	2020 km2 Population Grid Dataset
prec 1-12	January to December precipitation	alt	Altitude
tavg 1-12	January to December average temperature	slope	Slope
tmax 1-12	January to December maximum temperature	zbyl	Vegetation Classification
tmin 1-12	January to December minimum temperature	aspect	Aspect
bio1	Annual Mean Temperature	coarse	Coarse fragments
bio2	Mean Diurnal Range	sand	Sand
bio3	Isothermality	silt	Silt
bio4	Temperature Seasonality	clay	Clay
bio5	Max Temperature of Warmest Month	bulk	Bulk Density
bio6	Min Temperature of Coldest Month	ref_bulk	Reference Bulk Density
bio7	Temperature Annual Range	org_cbn	Organic Carbon Content
bio8	Mean Temperature of Wettest Quarter	ph	pH in water
bio9	Mean Temperature of Driest Quarter	n	Total nitrogen content
bio10	Mean Temperature of Warmest Quarter	cn	Carbon/Nitrogen ratio (C/N)
bio11	Mean Temperature of Coldest Quarter	cec_soil	CEC soil
bio12	Annual Precipitation	cec_clay	CEC clay
bio13	Precipitation of Wettest Month	teb	TEB
bio14	Precipitation of Driest Month	bsat	Base Saturation
bio15	Precipitation Seasonality	alum_sat	Aluminium saturation
bio16	Precipitation of Wettest Quarter	esp	Exchangeable Sodium Percentage
bio17	Precipitation of Driest Quarter	eq	Calcium Carbonate
bio18	Precipitation of Warmest Quarter	gypsum	Gypsum content
bio19	Precipitation of Coldest Quarter	elec_con	Electric Conductivity

**Figure 2:**
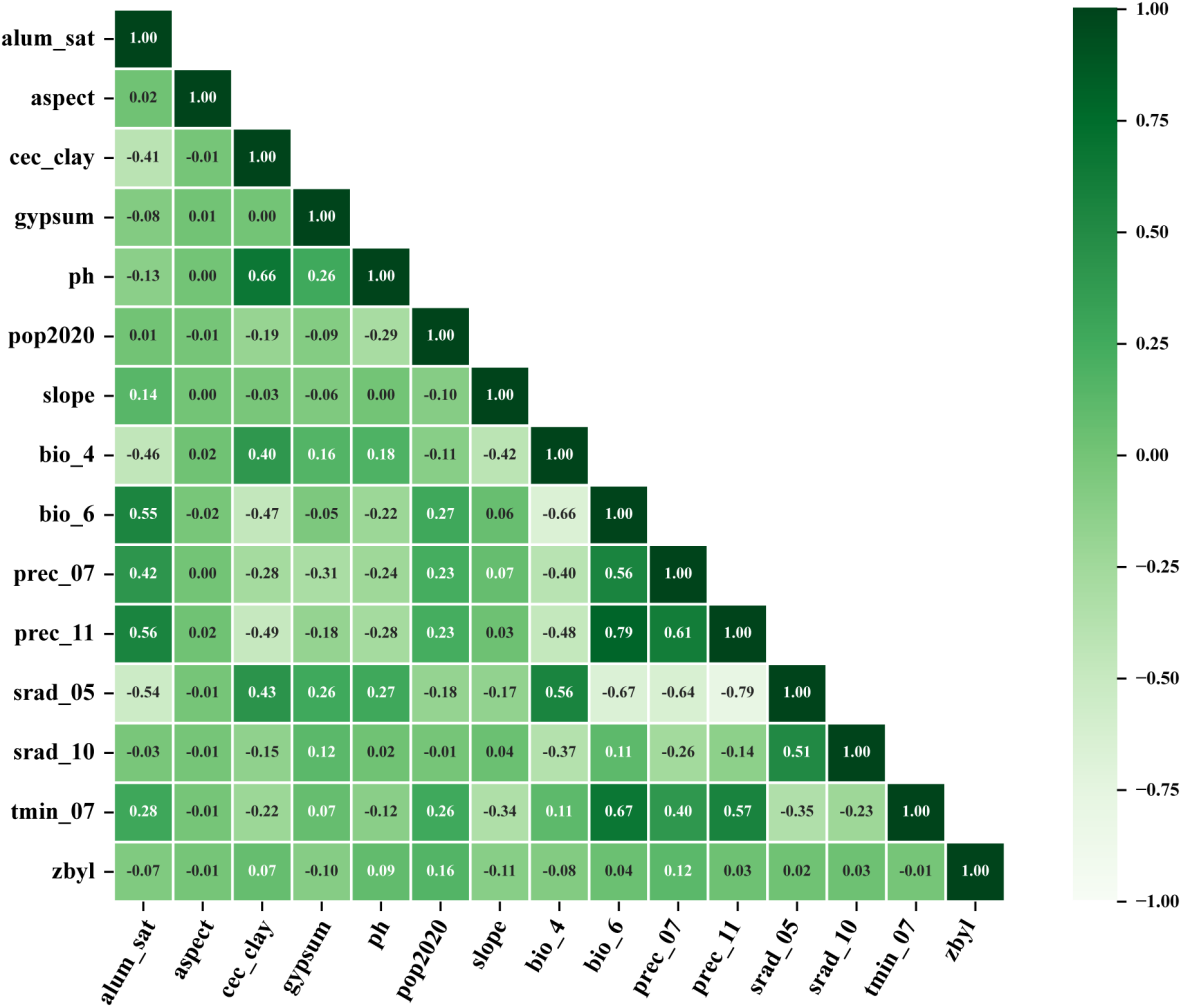
Pearson correlation matrix of the 15 environmental variables included in the model.

Because strong collinearity may occur among environmental variables (Dormann et al., 2013), key predictors were screened from the 103 candidate variables in three steps: (1) the 160 occurrence points and 103 environmental variables were jointly entered into MaxEnt to obtain the contribution of each variable to the preliminary model; (2) ENMTools was used to calculate pairwise Pearson correlation coefficients (|r|) among variables; and (3) for variable pairs with |r| ≥ 0.8, only the variable with higher contribution and clearer ecological meaning was retained. After this screening, 15 variables were retained for model construction, optimization, and evaluation: bio_6, srad_05, prec_11, bio_4, tmin_07, pop2020, zbyl, slope, prec_07, srad_10, cec_clay, ph, aspect, alum_sat, and gypsum. Correlations among the retained variables are shown in **Figure 2**, and the complete 103 × 103 correlation matrix is provided in the **Supplementary Material**.

### 2.3 MaxEnt model construction, optimization, and evaluation

The processed occurrence data and the 15 selected environmental variables were imported into MaxEnt 3.4.4 in CSV format. Model settings were as follows: 75% of the occurrence points were used for training and 25% for testing; bootstrap resampling was applied; the number of background points was set to 10,000; random seed was selected to improve reproducibility; 10 replicate runs were performed; and the output format was logistic.

Because the regularization multiplier (RM) and feature combination (FC) strongly affect MaxEnt predictions, the Kuenm package was used in R 4.4.1 to tune model parameters systematically (Muscarella et al., 2014; Cobos et al., 2019). Candidate feature types included linear, quadratic, hinge, product, and threshold features. RM ranged from 0.6 to 4.0 with an interval of 0.2, and 558 candidate parameter combinations were compared. The final model selected RM = 3.2 and a linear-product-threshold feature combination (LPT), which was then used for current and future suitability prediction.

Model accuracy was evaluated jointly using AUC, omission rate, and AICc. AUC is a receiver-operating-characteristic-based discrimination metric and should be interpreted together with other evaluation criteria (Parodi et al., 2022). Values of AUC closer to 1 indicate stronger discrimination ability, whereas omission rate and AICc reflect model error and model complexity, respectively. These metrics were therefore considered together to assess the predictive performance of the optimized model.

### 2.4 Data processing of MaxEnt outputs

To analyze the dynamics of suitable habitats under current and future scenarios, ArcGIS 10.8.1 was used for raster visualization and spatial statistics of MaxEnt outputs. The maximum test sensitivity plus specificity (MTSPS) threshold was used to classify suitable and unsuitable areas (Liu et al., 2005). The MTSPS value in this study was 0.182. Suitability was therefore divided into unsuitable (0-0.182), low suitability (0.182-0.5), medium suitability (0.5-0.7), and high suitability (0.7-1.0). The thresholds of 0.5 and 0.7 were used only for map classification and are not interpreted as physiological thresholds.

For area and spatial-change analysis, all classified rasters were projected by nearest-neighbor resampling to a 5 km China Albers equal-area grid, and area was calculated as cell count × 25 km^2^. The suitable habitat of *E. sagittatum* was further simplified into suitable and unsuitable classes. Current suitable habitat was overlaid with future suitable habitats for the 2050s and 2090s under SSP1-2.6, SSP2-4.5, and SSP5-8.5 to calculate stable suitable, expansion, and contraction areas. Geometric centroids were calculated in the equal-area coordinate system. The two WorldClim BCC-CSM2-MR bioclimatic files for SSP2-4.5 were downloaded on 25 December 2025 and used to update future bio_4 and bio_6 values in the 2050s and 2090s projections.

## 3 Results

### 3.1 Model optimization and accuracy evaluation

**Figure 3:**
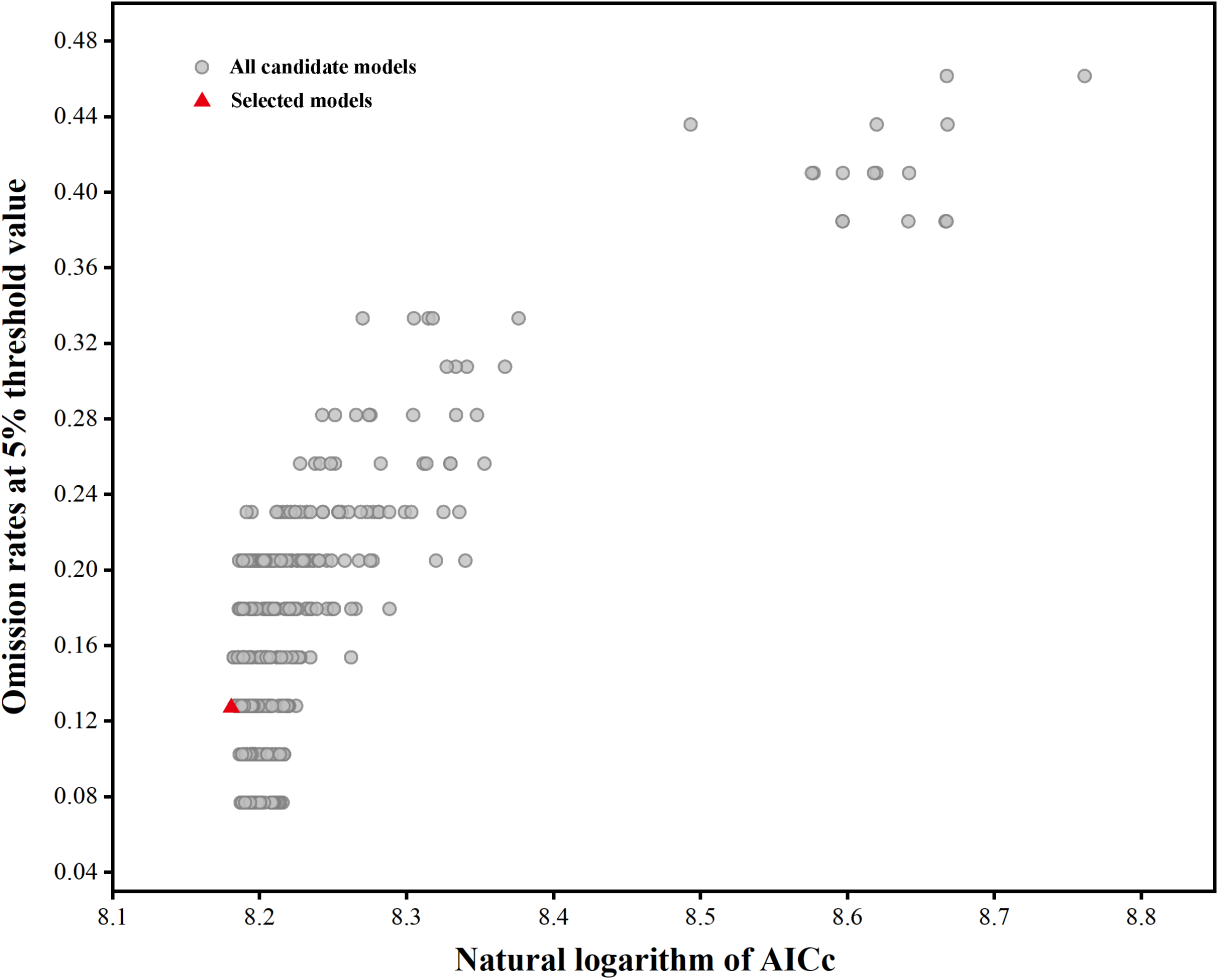
MaxEnt model parameter-optimization results.

In the MaxEnt model, Mean AUC Ratio represents predictive ability relative to random prediction, with higher values indicating stronger predictive performance; AICc reflects the trade-off between model fit and complexity, with lower values indicating lower overfitting risk while maintaining fit. Comparison between the default setting (FC = LQPH, RM = 1) and the Kuenm-recommended optimal setting (FC = LPT, RM = 3.2) showed that, under default parameters, Mean AUC Ratio was 1.840, AICc was 3823.26, and the 5% omission rate was 0.1795. Under the optimized parameters, Mean AUC Ratio decreased slightly, but AICc and omission rate decreased to 3579.69 and 0.1282, respectively, both clearly better than those of the default setting (**Figure 3**; **Table 2**). Considering these three metrics together, the optimized model maintained good discrimination while effectively reducing overfitting. Therefore, FC = LPT and RM = 3.2 were selected as the final modeling conditions for *E. sagittatum.*

**Table 2:** Performance evaluation of the MaxEnt model under default and optimized parameters.

Model evaluation	Feature combination	Regularization multiplier	Mean AUC Ratio	Omission rate at 5%	AICc
Default	LQPH	1	1.840	0.1795	3823.26
Optimized	LPT	3.2	1.814	0.1282	3579.69

### 3.2 Effects of key environmental variables on the distribution of *E. sagittatum*

Analysis of the 15 key environmental variables in the MaxEnt model showed that minimum temperature of the coldest month (bio_6), May solar radiation (srad_05), November precipitation (prec_11), and temperature seasonality (bio_4) were the four dominant environmental factors affecting the distribution of *E. sagittatum*. Their contributions were 33.4%, 20.3%, 18.3%, and 8.8%, respectively, and their cumulative contribution reached 80.8% (**Table 3**). Among them, bio_6 ranked first, indicating that winter low temperature is the primary climatic constraint on the distribution of *E. sagittatum*. Among the remaining 11 variables, tmin_07, pop2020, zbyl, and slope contributed 3.1%, 2.4%, 2.2%, and 2.1%, respectively, whereas all other variables contributed less than 2%. Together, these remaining variables accounted for only 19.2% and played a more limited role in constraining the overall model.

**Table 3:** Contribution and permutation importance of environmental variables.

Variable code	Environmental factor	Unit	Percent contribution/%	Permutation importance/%
bio_6	Min Temperature of Coldest Month	°C	33.4	11.4
srad_05	Solar Radiation in May	kJ·m-2·d-1	20.3	27.3
prec_11	Precipitation in November	mm	18.3	8.3
bio_4	Temperature Seasonality	°C×100	8.8	12.1
tmin_07	Min Temperature in July	°C	3.1	3.7
pop2020	Population Density	people/km2	2.4	1.4
zbyl	Vegetation Classification	-	2.2	3.0
slope	Slope	°	2.1	4.6
prec_07	Precipitation in July	mm	2.0	4.2
srad_10	Solar Radiation in October	kJ·m-2·d-1	1.9	2.9
cec_clay	CEC of Clay	cmolc/kg	1.6	6.0
ph	Soil pH	-	1.6	9.0
aspect	Aspect	rad	0.9	1.1
alum_sat	Aluminium Saturation	%	0.9	2.7
gypsum	Gypsum Content	%	0.3	2.2

**Figure 4:**
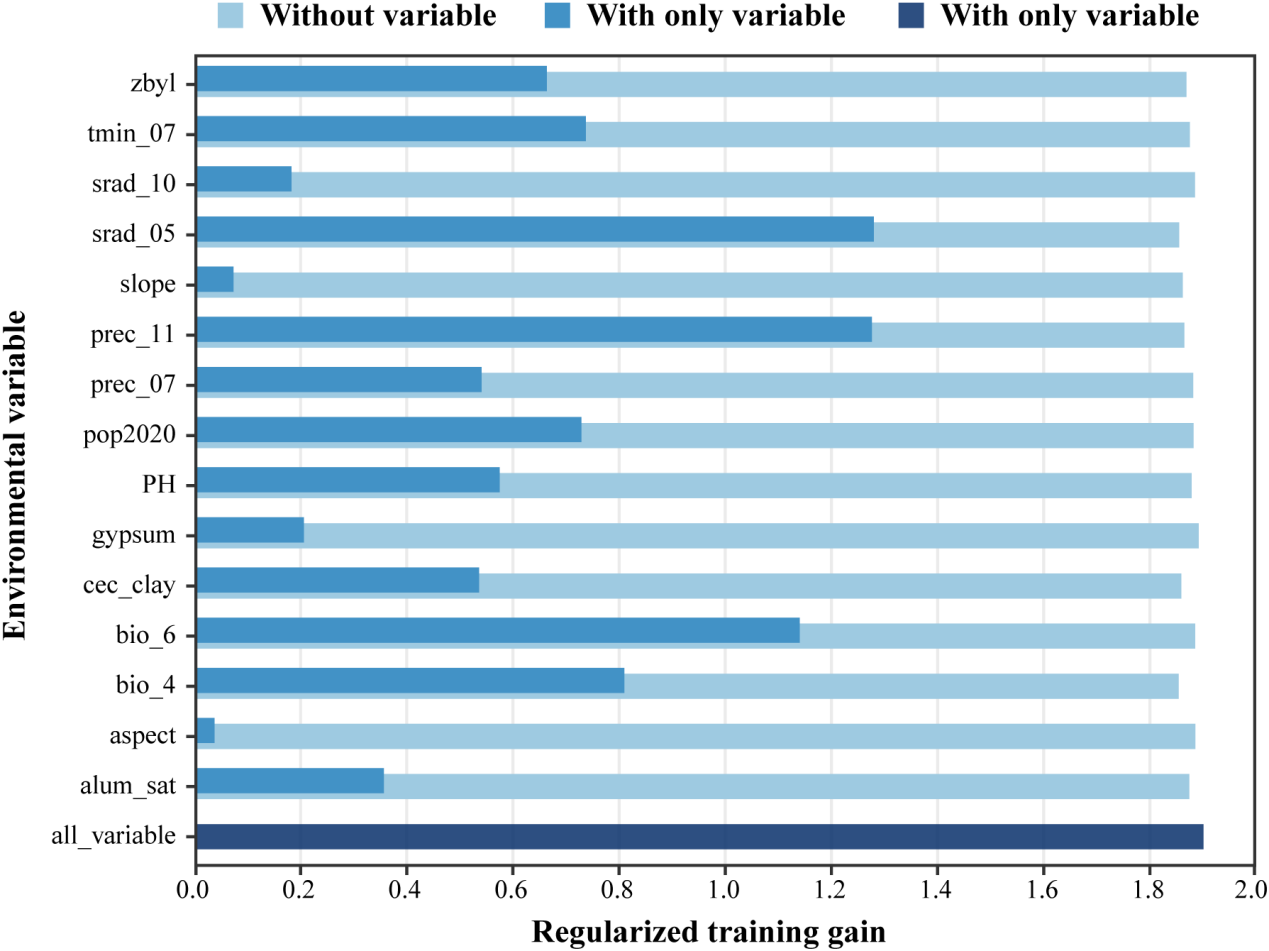
Jackknife test results for environmental-variable importance in the MaxEnt model.

The independent importance of each variable was further tested using the Jackknife method (**Figure 4**). When the model was run using a single environmental variable, bio_6, srad_05, prec_11, and bio_4 produced the highest regularized training gains, fully consistent with the dominant factors identified by contribution analysis. This result confirms the central role of these four variables in predicting suitable habitats of *E. sagittatum*.

**Figure 5:**
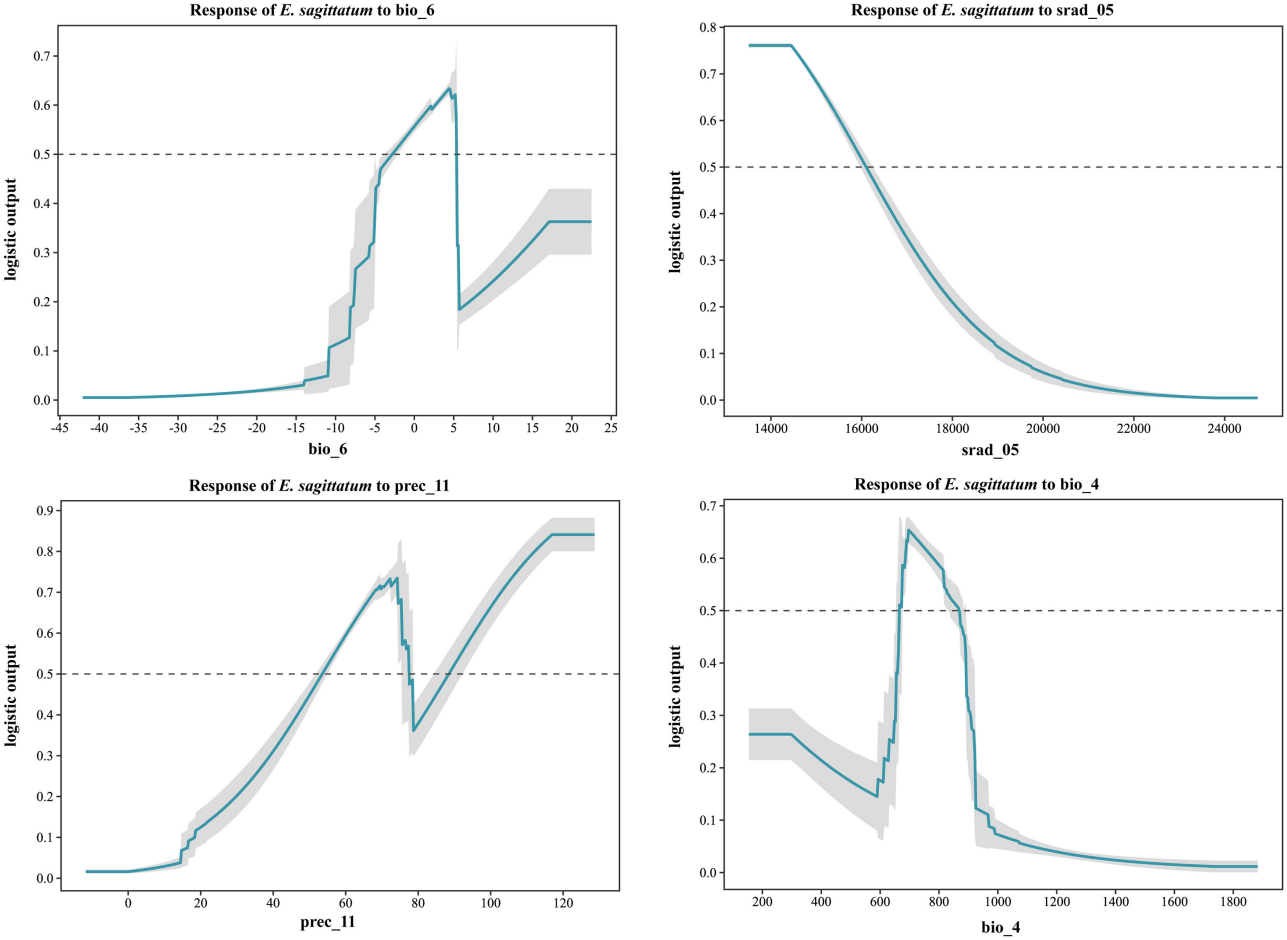
Response curves of dominant environmental variables (bio_4, bio_6, srad_05, and prec_11) for *E. sagittatum* suitability.

Response curves were generated for the dominant variables to examine their effects on suitability probability (**Figure 5**). Using a logistic output value of 0.5 as the suitability threshold, the curves showed that the suitable range of bio_6 (minimum temperature of the coldest month) was -2.75 to 5.33°C, the suitable range of srad_05 (May solar radiation) was 13,514 to 16,094 kJ m-2 d-1, and temperature seasonality (bio_4) showed a typical unimodal response with a suitable range of 665.86 to 868.16 (x 0.01°C). November precipitation (prec_11) showed a bimodal response, with suitable intervals of 53.59-77.45 mm and 88.55-128.70 mm. These thresholds define the main climatic niche of *E. sagittatum* in the modelled environmental space.

### 3.3 Suitable-habitat distribution of *E. sagittatum* under current climatic conditions

**Figure 6:**
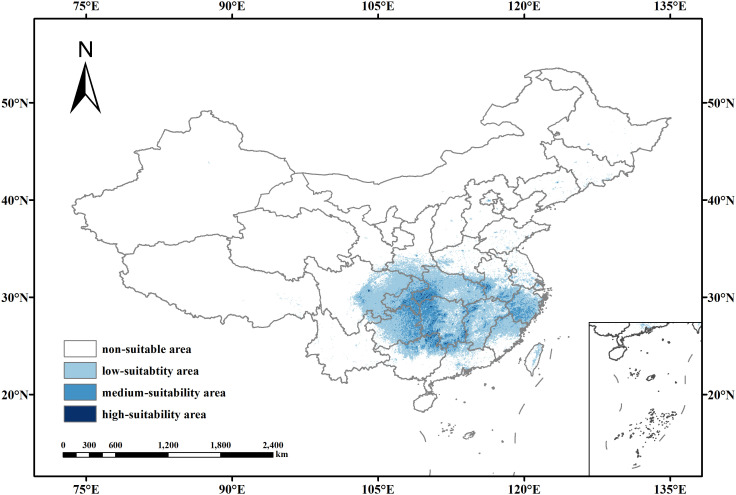
Potential suitable distribution of *E. sagittatum* under current climatic conditions.

Based on the MaxEnt outputs, the potential suitable habitat of *E. sagittatum* was classified into four levels using MTSPS = 0.182 as the threshold: unsuitable area (0-0.182), low-suitability area (0.182-0.5), medium-suitability area (0.5-0.7), and high-suitability area (0.7-1.0) (**Figure 6**). Under current climatic conditions, the total suitable area of *E. sagittatum* in China was 1.30 × 10^6^ km^2^, accounting for 13.51% of China’s land area. The suitable habitat extended mainly along the southern side of the middle Yangtze River and covered the core humid regions of central and eastern China.

The high-suitability area covered 1.21 × 10^4^ km^2^, accounting for 0.13% of the land area, and was concentrated in the low mountain and hilly region around the Dongting Lake-Poyang Lake area near the junction of northern Hunan, southern Hubei, northern Jiangxi, and southern Anhui. The medium-suitability area covered 2.86 × 10^5^ km^2^, accounting for 2.98%, and extended outward from the high-suitability core to include most of Hunan, southern Hubei, central and northern Jiangxi, central and southern Anhui, western Zhejiang, and mountainous areas of eastern Guizhou and northern Fujian. The low-suitability area covered 9.99 × 10^5^ km^2^, accounting for 10.40%, extending westward to the outer margin of the Sichuan Basin and much of Guizhou, southward to northern Guangdong and northern Guangxi, and northward to southern Shaanxi and southern Henan. Unsuitable areas mainly covered the arid northwest, the alpine Qinghai-Tibet Plateau, much of North China, Northeast China, and low-elevation coastal areas of South China.

### 3.4 Suitable-habitat distribution of *E. sagittatum* under future climatic conditions

**Figure 7:**
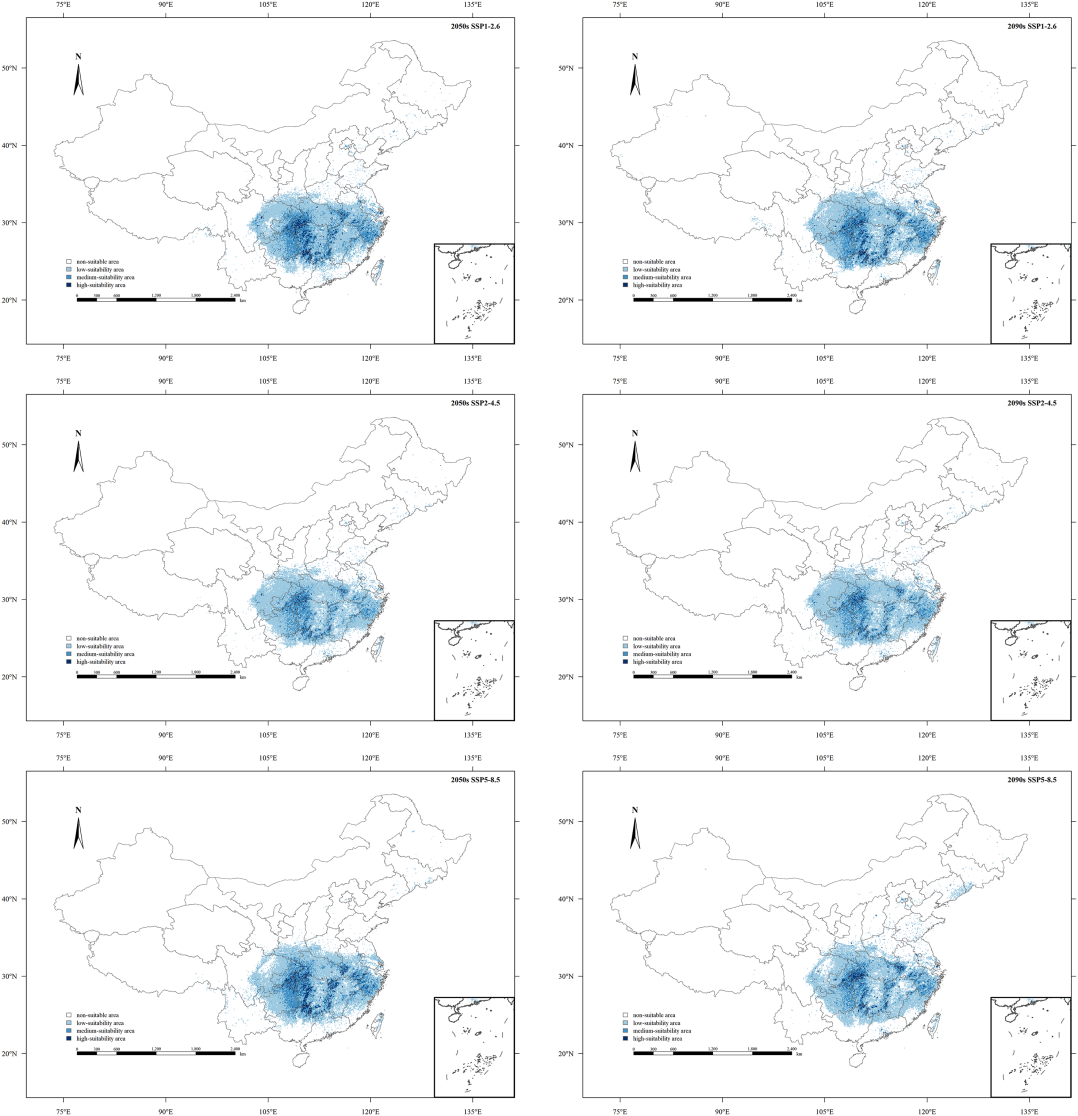
Potential suitable distribution of *E. sagittatum* in the 2050s and 2090s under three SSP pathways.

Based on the BCC-CSM2-MR model, potential suitable habitats were simulated for the 2050s (2041-2060) and 2090s (2081-2100) under SSP1-2.6, SSP2-4.5, and SSP5-8.5. Areas of different suitability levels are shown in **Table 4**, and spatial distributions are shown in **Figure 7**. Compared with current conditions, future total suitable area differed among periods and pathways and did not increase monotonically with emission intensity.

**Table 4:** Area of different suitability levels for *E. sagittatum* under current and future scenarios (× 10^4^ km^2^).

Scenario-period	Low	Medium	High	Total	Change (%)
Current	105.16	29.28	3.23	137.67	0.00
2050s SSP1-2.6	101.23	38.97	8.22	148.42	7.81
2050s SSP2-4.5	107.92	26.64	2.81	137.35	-0.23
2050s SSP5-8.5	103.92	37.48	9.50	150.90	9.61
2090s SSP1-2.6	99.51	37.52	6.74	143.76	4.43
2090s SSP2-4.5	110.26	27.96	3.19	141.40	2.71
2090s SSP5-8.5	106.88	29.83	5.80	142.51	3.52

In the 2050s, total suitable areas under SSP1-2.6, SSP2-4.5, and SSP5-8.5 were 1.48 × 10^6^, 1.37 × 10^6^, and 1.51 × 10^6^ km^2^, respectively, corresponding to changes of +7.81%, -0.23%, and +9.61% relative to the current area. The total suitable area under SSP2-4.5 in the 2050s was close to the current level, whereas SSP1-2.6 and SSP5-8.5 showed larger increases.

In the 2090s, total suitable areas under SSP1-2.6, SSP2-4.5, and SSP5-8.5 were 1.44 × 10^6^, 1.4140 × 10^6^, and 1.43 × 10^6^ km^2^, respectively, representing increases of 4.43%, 2.71%, and 3.52%. These results indicate clear scenario dependence in future suitable-habitat change, with different pathways producing different magnitudes of change.

### 3.5 Spatial pattern changes in the future potential distribution of *E. sagittatum*

**Figure 8:**
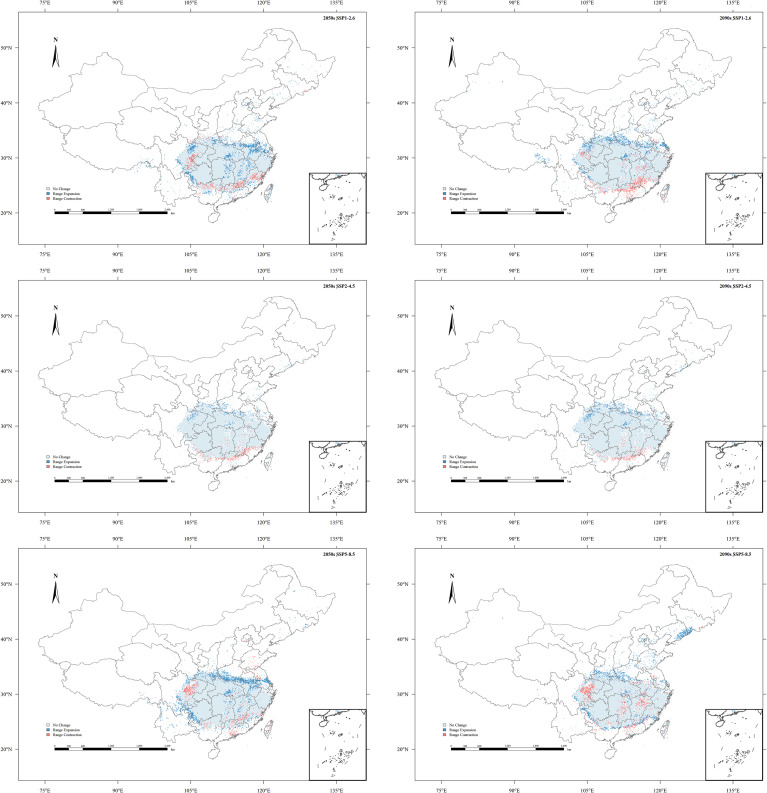
Stable, expansion, and contraction patterns of the potential suitable habitat of *E. sagittatum* under six future projections.

After overlaying suitable habitats under the six future scenario-period combinations with the current suitable habitat, changed areas were classified as stable suitable, expansion, and contraction areas. Stable suitable area dominated in all future projections, indicating a degree of persistence in the core suitable region of the model (**Figure 8**; **Table 5**).

**Table 5:** Stable suitable, expansion, and contraction areas under six future projections (× 10^4^ km^2^).

Scenario-period	Stable suitable	Expansion	Contraction	Net change
2050s SSP1-2.6	129.85	18.57	7.82	10.76
2050s SSP2-4.5	132.85	4.50	4.81	-0.31
2050s SSP5-8.5	129.85	21.05	7.81	13.23
2090s SSP1-2.6	128.70	15.07	8.97	6.10
2090s SSP2-4.5	134.05	7.35	3.62	3.73
2090s SSP5-8.5	127.27	15.24	10.40	4.84

Under SSP2-4.5, expansion and contraction areas in the 2050s were 4.50 × 10^4 and 4.81 × 10^4^ km^2^, respectively, with net change close to zero. In the 2090s, expansion area reached 7.35 × 10^4^ km^2^ and contraction area decreased to 3.62 × 10^4^ km^2^, producing a net increase of 3.73 × 10^4^ km^2^. This result is consistent with the moderate suitable-area change under the intermediate pathway.

Overall, future expansion areas were mainly located along the northern margin of the current suitable habitat, whereas contraction areas occurred more often along the southern margin and in locally fragmented areas. This northward tendency is consistent with many studies of plant distributions under climate change, and it indicates that resource monitoring and field surveys should pay attention to the northern transition zone of the current suitable range.

### 3.6 Centroid migration of suitable habitats

**Figure 9:**
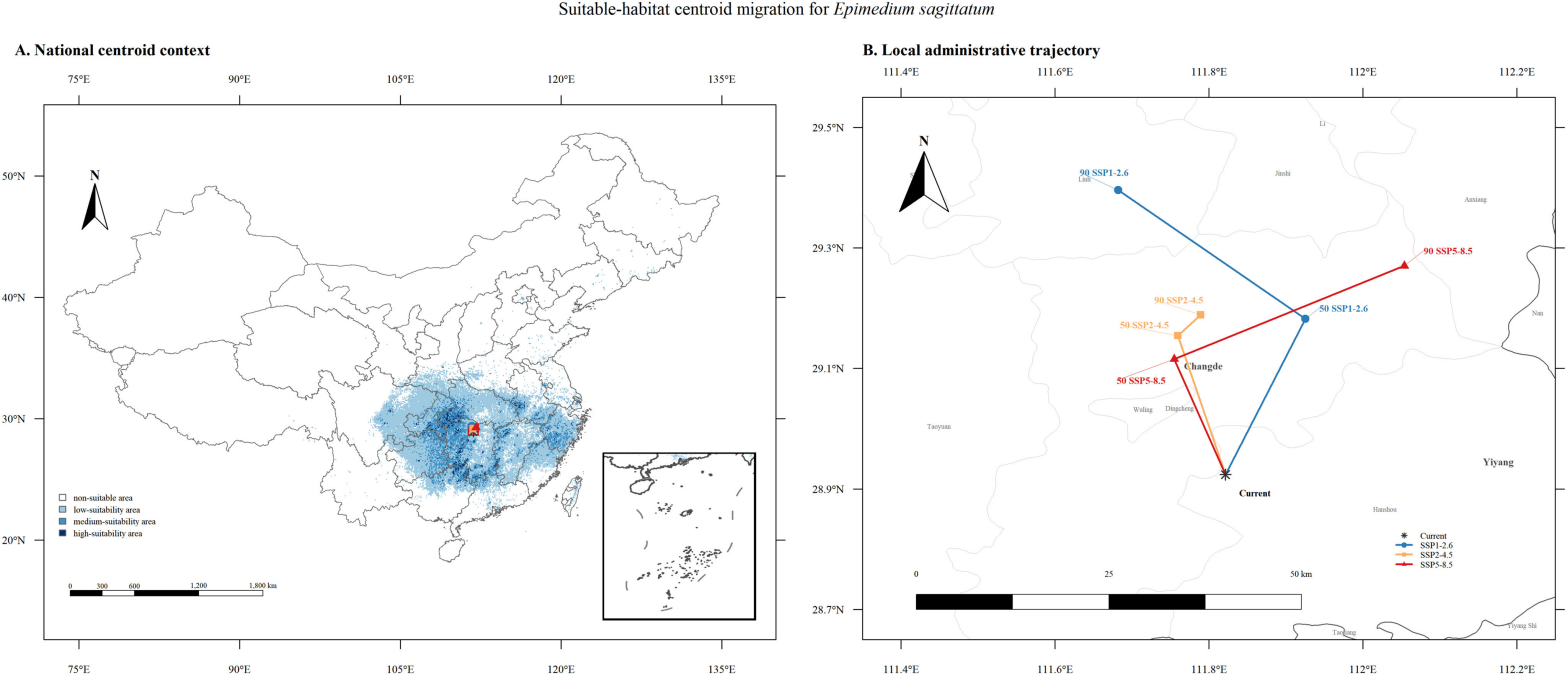
Geometric-centroid migration trajectory of suitable habitats of *E. sagittatum* under current and future climate scenarios.

Using MTSPS = 0.182 as the threshold, the study area was divided into suitable and unsuitable regions, and the geometric centroid of the suitable habitat was calculated in an equal-area projection (**Figure 9**; **Table 6**). Under current climatic conditions, the centroid of the suitable habitat of *E. sagittatum* was located at 111.822°E and 28.924°N, near Changde, Hunan Province. This position is close to the middle Yangtze River humid region, where the current suitable area is concentrated, and can therefore be used as the baseline for evaluating future spatial displacement.

**Table 6:** Centroids of suitable habitats and displacement relative to the current centroid.

Scenario-period	Longitude/°E	Latitude/°N	Displacement/km	Bearing/°
Current	111.822	28.924	0.0	0.0
2050s SSP1-2.6	111.925	29.182	30.6	15.1
2050s SSP2-4.5	111.760	29.154	26.5	343.1
2050s SSP5-8.5	111.755	29.115	22.4	339.4
2090s SSP1-2.6	111.682	29.396	54.5	341.9
2090s SSP2-4.5	111.789	29.189	29.8	350.1
2090s SSP5-8.5	112.054	29.270	44.8	26.0

Relative to the current centroid, future centroids shifted by 22.4-54.5 km. In the 2050s, centroid displacement was 30.6 km under SSP1-2.6, 26.5 km under SSP2-4.5, and 22.4 km under SSP5-8.5. In the 2090s, displacement increased to 54.5 km under SSP1-2.6, 29.8 km under SSP2-4.5, and 44.8 km under SSP5-8.5. The largest displacement occurred under SSP1-2.6 in the 2090s, whereas the smallest occurred under SSP5-8.5 in the 2050s. These values show that the centroid of suitable habitat moved within a relatively short distance compared with the national study extent.

The direction of centroid movement differed among scenarios, but most future centroids showed a northward component. Under SSP1-2.6, the centroid moved slightly northeastward in the 2050s and northwestward by the 2090s; under SSP2-4.5, movement was mainly north to northwest, with a relatively small displacement; under SSP5-8.5, the centroid shifted northwestward in the 2050s and northeastward in the 2090s. Together with the expansion and contraction analysis, these trajectories suggest that future suitable habitats may expand more along the northern margin while the southern margin becomes more fragmented. However, the limited displacement distance also indicates that the overall distribution center remains anchored around the current core suitable region.

## 4 Discussion

Ecological niche models infer species’ ecological requirements through algorithms and use them to predict potential distributions; therefore, improving model accuracy is essential for reliable suitability assessment. To predict the suitable habitats of *E. sagittatum* more accurately, this study optimized MaxEnt from three aspects: occurrence-data processing, environmental-variable screening, and model-parameter tuning. First, ENMTools was used to remove duplicate or neighboring occurrence points within 5 km × 5 km grids, reducing spatially correlated samples and sampling bias. Second, variables with high collinearity (|r| >= 0.8) were removed from the 103 candidate environmental factors based on Pearson correlations calculated in ENMTools and variable contributions from MaxEnt, leaving 15 key variables with clear ecological meaning and reducing the overfitting risk caused by redundant predictors (Dormann et al., 2013). Finally, Kuenm was used to systematically search 558 parameter combinations (Muscarella et al., 2014; Cobos et al., 2019), and the optimal setting was FC = LPT and RM = 3.2. After optimization, AICc decreased from 3823.26 to 3579.69 and the 5% omission rate also decreased, indicating that the model had relatively high reliability for predicting suitable habitats of *E. sagittatum*.

The model identified bio_6, srad_05, prec_11, and bio_4 as the main environmental variables affecting the distribution of *E. sagittatum*. The importance of minimum temperature of the coldest month indicates that winter low temperature may limit the northern or high-elevation margins of the species. May solar radiation may be related to spring growth of this understory plant. November precipitation may influence late-autumn soil moisture and overwintering habitat quality. Temperature seasonality suggests an association with relatively stable subtropical thermal conditions. These explanations are ecologically reasonable, but they still need to be tested through physiological experiments and field observations.

The predicted current suitable area is broadly consistent with the distribution described in Flora of China, especially in Hunan, Hubei, Jiangxi, Anhui, Zhejiang, and adjacent humid mountainous regions (Ying et al., 2011). This agreement indicates that the model can help identify priority areas for resource conservation and field surveys. Low-suitability areas outside the known distribution range may serve as supplementary survey areas, whereas medium- and high-suitability areas provide stronger spatial references for germplasm conservation and cultivation-zoning evaluation. If these predictions are used for cultivation zoning, they should be combined with land use, soil management, understory shading, provenance, and field-trial results.

Future projections show that suitable habitats of *E. sagittatum* remain concentrated mainly in humid central and eastern China, with northward expansion along the suitable-area margin under some scenarios. SSP1-2.6 and SSP5-8.5 showed larger increases in the 2050s, whereas SSP2-4.5 showed almost no net change in the 2050s and only a moderate increase in the 2090s. The centroid shifted generally northward, but the distance was limited, indicating that climate change may add some marginal suitable areas while the priority for conservation and management should still remain in the current and nearby core suitable regions. Similar plant and medicinal-plant suitability studies have reported upward shifts, range expansion or contraction, and conservation-priority changes under future climates (Lenoir et al., 2008; Rana et al., 2020; Zou et al., 2023).

This study has practical value for medicinal-plant resource conservation and zoning. First, the environmental variables covered climate, topography, vegetation, population density, and soil properties, allowing the formation of potential suitable habitats to be interpreted from multiple environmental dimensions. Second, the future analysis compared low-, intermediate-, and high-emission pathways for two representative periods, the 2050s and 2090s. Third, all suitable-area and change-area statistics were calculated under an equal-area projection, making the area estimates more reliable. Fourth, the map series presents current suitability, future suitability, range change, and centroid migration in a unified framework, providing intuitive spatial evidence for conservation surveys and cultivation-zoning discussion.

Future research should further incorporate field-verified occurrence records, spatially structured validation, multi-GCM comparison, ensemble modeling, and variables such as land-use change, understory microhabitat, harvesting pressure, biotic interactions, and dispersal capacity. Combining model predictions with field trials and resource surveys will help transform potential suitability maps into more operational conservation and cultivation-zoning schemes.

## 5 Conclusion

Based on 160 valid occurrence points of *E. sagittatum* and 103 environmental variables, this study used Kuenm to optimize the feature combination and regularization multiplier of the MaxEnt model and systematically predicted the current and future potential suitable distribution and dynamic changes of *E. sagittatum* in China. The results showed that the distribution of *E. sagittatum* was mainly associated with winter temperature, spring radiation, late-autumn precipitation, and temperature seasonality, with bio_6 contributing most strongly to the model.

Under current climatic conditions, suitable habitats of *E. sagittatum* were mainly distributed in the middle and lower Yangtze River region and adjacent humid areas, including Hunan, Hubei, Jiangxi, Anhui, and Zhejiang, with a total suitable area of 1.38 × 10^6^ km^2^. Under future scenarios, total suitable area ranged from 1.37 × 10^6^ to 1.51 × 10^6^ km^2^ in the 2050s and from 1.41 × 10^^6^ to 1.44 × 10^6^ km^2^ in the 2090s. Expansion areas were generally located along the northern margin of the current suitable habitat, contraction occurred more often along the southern margin, and centroid movement was generally northward but limited in distance.

These results provide a spatial reference for *in-situ* conservation, resource surveys, and cultivation zoning of *E. sagittatum* under climate change. Future integration of field validation and multi-source environmental assessment will further support the practical application of suitability predictions in resource conservation and standardized cultivation.

## Data Availability

The original contributions presented in the study are included in the article/**Supplementary Material**. Further inquiries can be directed to the corresponding authors.

## References

[B1] Anderson R. P. (2013). A framework for using niche models to estimate impacts of climate change on species distributions. Ann. N. Y. Acad. Sci. 1297, 8–28. doi: 10.1111/nyas.12264 25098379

[B2] Applequist W. L. Brinckmann J. A. Cunningham A. B. Hart R. E. Heinrich M. Katerere D. R. . (2020). Scientists' warning on climate change and medicinal plants. Planta Med. 86, 10–18. doi: 10.1055/a-1041-3406 31731314

[B3] Bi Z. Zhang W. Yan X. (2022). Anti-inflammatory and immunoregulatory effects of icariin and icaritin. Biomedicine Pharmacotherapy 151, 113180. doi: 10.1016/j.biopha.2022.113180 35676785

[B4] Boria R. A. Olson L. E. Goodman S. M. Anderson R. P. (2014). Spatial filtering to reduce sampling bias can improve the performance of ecological niche models. Ecol. Modell. 275, 73–77. doi: 10.1016/j.ecolmodel.2013.12.012 38826717

[B5] Chen S. Jiang Z. Song J. Xie T. Xue Y. Yang Q. (2025). Prediction of potential habitat of Verbena officinalis in China under climate change based on optimized MaxEnt model. Front. Plant Sci. 16, 1563070. doi: 10.3389/fpls.2025.1563070 40177015 PMC11961872

[B6] Cheng L. Jin X. Shen H. Chen X. Chen J. Xu B. . (2022). Icariin attenuates thioacetamide-induced bone loss via the RANKL-p38/ERK-NFAT signaling pathway. Mol. Med. Rep. 25, 126. doi: 10.3892/mmr.2022.12642 35169865 PMC8864607

[B7] Cobos M. E. Peterson A. T. Barve N. Osorio-Olvera L. (2019). kuenm: an R package for detailed development of ecological niche models using Maxent. PeerJ 7, e6281. doi: 10.7717/peerj.6281 30755826 PMC6368831

[B8] Dormann C. F. Elith J. Bacher S. Buchmann C. Carl G. Carré G. (2013). Collinearity: a review of methods to deal with it and a simulation study evaluating their performance. Ecography 36, 27–46. doi: 10.1111/j.1600-0587.2012.07348.x 40046247

[B9] Elith J. Graham C. H. Anderson R. P. Dudík M. Ferrier S. Guisan A. (2006). Novel methods improve prediction of species' distributions from occurrence data. Ecography 29, 129–151. doi: 10.1111/j.2006.0906-7590.04596.x 40046247

[B10] Elith J. Phillips S. J. Hastie T. Dudík M. Chee Y. E. Yates C. J. (2011). A statistical explanation of MaxEnt for ecologists. Divers. Distrib. 17, 43–57. doi: 10.1111/j.1472-4642.2010.00725.x 40046247

[B11] FAO/IIASA/ISRIC/ISS-CAS/JRC (2012). Harmonized World Soil Database (Version 1.2), FAO/IIASA/ISRIC/ISS-CAS/JRC (2012). Harmonized World Soil Database (version 1.2). FAO, Rome, and IIASA, Laxenburg.

[B12] Fick S. E. Hijmans R. J. (2017). WorldClim 2: new 1-km spatial resolution climate surfaces for global land areas. Int. J. Climatol. 37, 4302–4315. doi: 10.1002/joc.5086 41531421

[B13] Hijmans R. J. Cameron S. E. Parra J. L. Jones P. G. Jarvis A. (2005). Very high resolution interpolated climate surfaces for global land areas. Int. J. Climatol. 25, 1965–1978. doi: 10.1002/joc.1276 41531421

[B14] Lenoir J. Gégout J. C. Marquet P. A. De Ruffray P. Brisse H. (2008). A significant upward shift in plant species optimum elevation during the 20th century. Science 320, 1768–1771. doi: 10.1126/science.1156831 18583610

[B15] Liu C. Berry P. M. Dawson T. P. Pearson R. G. (2005). Selecting thresholds of occurrence in the prediction of species distributions. Ecography 28, 385–393. doi: 10.1111/j.0906-7590.2005.03957.x 40046247

[B16] Ma D. Lun X. Li C. Zhou R. Zhao Z. Wang J. (2021). Predicting the potential global distribution of Amblyomma americanum (Acari: Ixodidae) under near current and future climatic conditions, using the maximum entropy model. Biology 10, 1057. doi: 10.3390/biology10101057 34681156 PMC8533137

[B17] Merow C. Smith M. J. Silander J. A. (2013). A practical guide to MaxEnt for modeling species' distributions: what it does, and why inputs and settings matter. Ecography 36, 1058–1069. doi: 10.1111/j.1600-0587.2013.07872.x 40046247

[B18] Muscarella R. Galante P. J. Soley-Guardia M. Boria R. A. Kass J. M. Uriarte M. (2014). ENMeval: An R package for conducting spatially independent evaluations and estimating optimal model complexity for Maxent ecological niche models. Methods Ecol. Evol. 5, 1198–1205. doi: 10.1111/2041-210X.12261 40046247

[B19] O'Neill B. C. Tebaldi C. van Vuuren D. P. Eyring V. Friedlingstein P. Hurtt G. . (2016). The scenario model intercomparison project (ScenarioMIP) for CMIP6. Geosci. Model. Dev. 9, 3461–3482. doi: 10.5194/gmd-9-3461-2016

[B20] Parmesan C. Yohe G. (2003). A globally coherent fingerprint of climate change impacts across natural systems. Nature 421, 37–42. doi: 10.1038/nature01286 12511946

[B21] Parodi S. Verda D. Bagnasco F. Muselli M. (2022). The clinical meaning of the area under a receiver operating characteristic curve for the evaluation of the performance of disease markers. Epidemiol. Health 44, e2022088. doi: 10.4178/epih.e2022088 36265519 PMC10089712

[B22] Pearson R. G. Raxworthy C. J. Nakamura M. Peterson A. T. (2007). Predicting species distributions from small numbers of occurrence records: a test case using cryptic geckos in Madagascar. J. Biogeogr. 34, 102–117. doi: 10.1111/j.1365-2699.2006.01594.x 40046247

[B23] Phillips S. J. Anderson R. P. Schapire R. E. (2006). Maximum entropy modeling of species geographic distributions. Ecol. Modell. 190, 231–259. doi: 10.1016/j.ecolmodel.2005.03.026 38826717

[B24] Rana S. K. Rana H. K. Ranjitkar S. Ghimire S. K. Gurmachhan C. M. O'Neill A. R. . (2020). Climate-change threats to distribution, habitats, sustainability, and conservation of highly traded medicinal and aromatic plants in Nepal. Ecol. Indic. 115, 106435. doi: 10.1016/j.ecolind.2020.106435 38826717

[B25] Rong W. Huang X. Hu S. Zhang X. Jiang P. Niu P. (2024). Impacts of climate change on the habitat suitability and natural product accumulation of the medicinal plant Sophora alopecuroides L. based on the MaxEnt model. Plants 13, 1424. doi: 10.3390/plants13111424 38891233 PMC11174999

[B26] Shen T. Yu H. Wang Y. Z. (2021). Assessing the impacts of climate change and habitat suitability on the distribution and quality of medicinal plant using multiple information integration: take Gentiana rigescens as an example. Ecol. Indic. 123, 107376. doi: 10.1016/j.ecolind.2021.107376 38826717

[B27] Shi X. Wang J. Zhang L. Chen S. Zhao A. Ning X. (2023). Prediction of the potentially suitable areas of Litsea cubeba in China based on future climate change using the optimized MaxEnt model. Ecol. Indic. 148, 110093. doi: 10.1016/j.ecolind.2023.110093 37869439 PMC10585429

[B28] Wan J. Wang R. Ren Y. Mckirdy S. (2020). Potential distribution and the risks of Bactericera cockerelli and its associated plant pathogen Candidatus Liberibacter solanacearum for global potato production. Insects 11, 298. doi: 10.3390/insects11050298 32408479 PMC7291056

[B29] Wang H. Sun K. Tan W. S. Gao J. Yuan L. Wen J. . (2025). Icariin promoted ferroptosis by activating mitochondrial dysfunction to inhibit colorectal cancer and synergistically enhanced the efficacy of PD-1 inhibitors. Phytomedicine 136, 156224. doi: 10.1016/j.phymed.2024.156224 39642461

[B30] WorldPop (2020). Global High-Resolution Population Denominators Project. Available online at: https://www.worldpop.org/ (Accessed November 30, 2025).

[B31] Wu T. Lu Y. Fang Y. Xin X. Li L. Li W. . (2019). The Beijing Climate Center Climate System Model (BCC-CSM): the main progress from CMIP5 to CMIP6. Geosci. Model. Dev. 12, 1573–1600. doi: 10.5194/gmd-12-1573-2019

[B32] Xi S. Guo X. Ma X. Jin L. (2025). Impacts of climate change on the suitable habitat of Angelica sinensis and analysis of its drivers in China. Sci. Rep. 15, 3508. doi: 10.1038/s41598-025-87436-3 39875443 PMC11775104

[B33] Ying J. S. Boufford D. E. Brach A. R. (2011). Flora of China Vol. 19. Eds. Wu Z. Y. Raven P. H. Hong D. Y. (Beijing; St. Louis: Science Press; Missouri Botanical Garden Press), 787–799.

[B34] Zhang K. Yao L. Meng J. Tao J. (2018). MaxEnt modeling for predicting the potential geographical distribution of two peony species under climate change. Sci. Total Environ. 634, 1326–1334. doi: 10.1016/j.scitotenv.2018.04.112 29710632

[B35] Zou H. Chen B. Zhang B. Zhou X. Zhang X. Zhang X. (2023). Conservation planning for the endemic and endangered medicinal plants under the climate change and human disturbance: a case study of Gentiana manshurica in China. Front. Plant Sci. 14, 1184556. doi: 10.3389/fpls.2023.1184556 37564387 PMC10410459

